# Bis{1-[(4-methyl­phen­yl)imino­meth­yl]-2-naphtho­lato-κ^2^ *N*,*O*}nickel(II)

**DOI:** 10.1107/S1600536811010087

**Published:** 2011-03-23

**Authors:** Quanbo Wang, Jianzhuang Jiang, Peihua Zhu

**Affiliations:** a School of Chemistry & Chemical Engineering, Shandong University, Jinan 250100, People’s Republic of China

## Abstract

In the title complex, [Ni(C_18_H_14_NO)_2_], the Ni^II^ ion lies on an inversion center and is coordinated in a slightly distorted square-planar environment. The 1-[(4-methyl­phen­yl)imino­meth­yl]-2-naphtho­late ligands are coordinated in a *trans* arrangement with respect to the N and O atoms. In the symmetry-unique ligand, the dihedral angle between the naphthalene ring system and the benzene ring of the methyl­phenyl group is 49.03 (7)°.

## Related literature

For the isostructural Cu analog and background information, see: Zhu *et al.* (2010 ▶). For a related Ni structure, see: Chang *et al.* (2004 ▶).
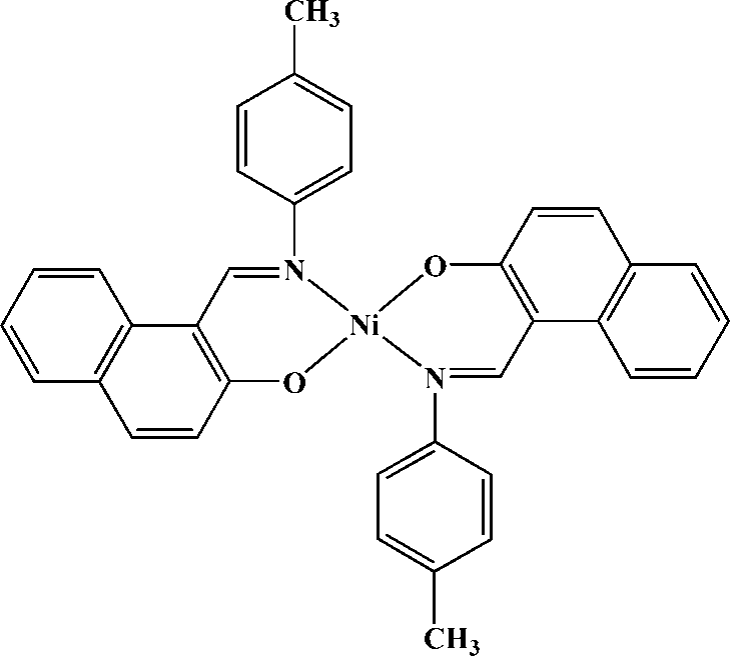


## Experimental

### 

#### Crystal data

[Ni(C_18_H_14_NO)_2_]
*M* *_r_* = 579.31
Triclinic, 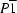

*a* = 7.1159 (4) Å
*b* = 9.9950 (5) Å
*c* = 10.5803 (5) Å
α = 103.057 (4)°
β = 96.327 (4)°
γ = 103.488 (4)°
*V* = 702.21 (6) Å^3^
*Z* = 1
Mo *K*α radiation
μ = 0.73 mm^−1^
*T* = 293 K
0.46 × 0.36 × 0.14 mm

#### Data collection

Bruker SMART CCD diffractometer
Absorption correction: multi-scan (*SADABS*; Sheldrick, 1996 ▶) *T*_min_ = 0.848, *T*_max_ = 1.0
6948 measured reflections
2753 independent reflections
2438 reflections with *I* > 2σ(*I*)
*R*_int_ = 0.023

#### Refinement

*R*[*F* ^2^ > 2σ(*F* ^2^)] = 0.029
*wR*(*F* ^2^) = 0.074
*S* = 1.07
2753 reflections
188 parameters
H-atom parameters constrained
Δρ_max_ = 0.21 e Å^−3^
Δρ_min_ = −0.19 e Å^−3^

### 

Data collection: *SMART* (Bruker, 2004 ▶); cell refinement: *SAINT* (Bruker, 2004 ▶); data reduction: *SAINT*; program(s) used to solve structure: *SHELXS97* (Sheldrick, 2008 ▶); program(s) used to refine structure: *SHELXL97* (Sheldrick, 2008 ▶); molecular graphics: *SHELXTL* (Sheldrick, 2008 ▶); software used to prepare material for publication: *SHELXTL*.

## Supplementary Material

Crystal structure: contains datablocks global, I. DOI: 10.1107/S1600536811010087/lh5218sup1.cif

Structure factors: contains datablocks I. DOI: 10.1107/S1600536811010087/lh5218Isup2.hkl

Additional supplementary materials:  crystallographic information; 3D view; checkCIF report

## Figures and Tables

**Fig. 1.**
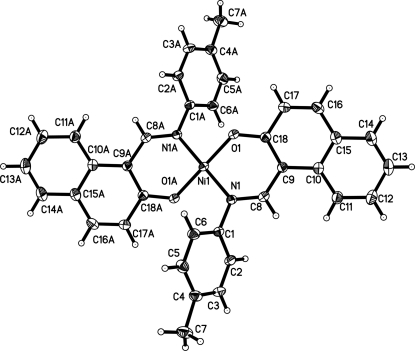
The molecular structure of (I). Displacement ellipsoids are drawn at the 30% probability level (symmetry code (A): -x+2, -y, -z).

## supplementary crystallographic information

### Comment

In a previous paper, we reported the crystal structure of Bis{1-[(4-methylphenyl)iminomethyl]-2-naphtholato-κ^2^ N,*O*}copper(II) (Zhu *et al.*, 2010). As part of our search for Schiff base metal complexes, the title compound, (I) (Fig. 1), was synthesized and its crystal structure is reported herein. The Ni^II^ ion is coordinated by two O atoms and two N atoms of two bidentate schiff base ligands to form a slightly distorted square-planar geometry with a *trans* arrangement. In the symmetry unique ligand the dihedral angle between the naphthalene ring [C9-C18] system and the benzene ring [C1-C6] of the methylphenyl group is 49.03 (7)°. The Ni—N and Ni—O bond lengths agree with those in a related complex (Chang *et al.*, 2004).

### Experimental

Nickel(II) acetate hydrate (0.194 g, 0.001 mol) in methanol (50 ml) and *N*-(*p*-Tolyl)-2-hydroxy-1-naphthaldimine (0.586 g, 0.002 mol) in acetonitrile(75 ml) were mixed and heated at 333 K for 1 h. The solution was filtered and the filtrate kept in a beaker at room temperature for crystallization. Needle-shaped crystals started appearing after 3 days.

### Refinement

Hydrogen atoms were placed in calculated positions and refined using a riding-model approximation with C—H = 0.93 Å, *U*_iso_ = 1.2*U*_eq_(C) for aromatic H atoms and C—H = 0.96 Å, *U*_iso_ = 1.5*U*_eq_(C) for methyl H atoms.

### Crystal data

**Table:**

[Ni(C_18_H_14_NO)_2_]	*Z* = 1
*M**_r_* = 579.31	*F*(000) = 302
Triclinic, *P*1	*D*_x_ = 1.370 Mg m^−^^3^
Hall symbol: -P 1	Mo *K*α radiation, λ = 0.71073 Å
*a* = 7.1159 (4) Å	Cell parameters from 4766 reflections
*b* = 9.9950 (5) Å	θ = 3.2–28.8°
*c* = 10.5803 (5) Å	µ = 0.73 mm^−^^1^
α = 103.057 (4)°	*T* = 293 K
β = 96.327 (4)°	Needle, colourless
γ = 103.488 (4)°	0.46 × 0.36 × 0.14 mm
*V* = 702.21 (6) Å^3^	

### Data collection

**Table:**

Bruker SMART CCD diffractometer	2753 independent reflections
Radiation source: fine-focus sealed tube	2438 reflections with *I* > 2σ(*I*)
graphite	*R*_int_ = 0.023
Detector resolution: 16.0355 pixels mm^-1^	θ_max_ = 26.0°, θ_min_ = 3.2°
ω scans	*h* = −8→8
Absorption correction: multi-scan (*SADABS*; Sheldrick, 1996)	*k* = −12→12
*T*_min_ = 0.848, *T*_max_ = 1.0	*l* = −12→13
6948 measured reflections	

### Refinement

**Table:**

Refinement on *F*^2^	Primary atom site location: structure-invariant direct methods
Least-squares matrix: full	Secondary atom site location: difference Fourier map
*R*[*F*^2^ > 2σ(*F*^2^)] = 0.029	Hydrogen site location: inferred from neighbouring sites
*wR*(*F*^2^) = 0.074	H-atom parameters constrained
*S* = 1.07	*w* = 1/[σ^2^(*F*_o_^2^) + (0.0426*P*)^2^] where *P* = (*F*_o_^2^ + 2*F*_c_^2^)/3
2753 reflections	(Δ/σ)_max_ = 0.001
188 parameters	Δρ_max_ = 0.21 e Å^−^^3^
0 restraints	Δρ_min_ = −0.19 e Å^−^^3^

### Special details

**Table:**

Geometry. All e.s.d.'s (except the e.s.d. in the dihedral angle between two l.s. planes) are estimated using the full covariance matrix. The cell e.s.d.'s are taken into account individually in the estimation of e.s.d.'s in distances, angles and torsion angles; correlations between e.s.d.'s in cell parameters are only used when they are defined by crystal symmetry. An approximate (isotropic) treatment of cell e.s.d.'s is used for estimating e.s.d.'s involving l.s. planes.
Refinement. Refinement of *F*^2^ against ALL reflections. The weighted *R*-factor *wR* and goodness of fit *S* are based on *F*^2^, conventional *R*-factors *R* are based on *F*, with *F* set to zero for negative *F*^2^. The threshold expression of *F*^2^ > σ(*F*^2^) is used only for calculating *R*-factors(gt) *etc*. and is not relevant to the choice of reflections for refinement. *R*-factors based on *F*^2^ are statistically about twice as large as those based on *F*, and *R*- factors based on ALL data will be even larger.

### Fractional atomic coordinates and isotropic or equivalent isotropic displacement parameters (Å^2^)

**Table:**

	*x*	*y*	*z*	*U*_iso_*/*U*_eq_	
Ni1	1.0000	0.0000	0.0000	0.03301 (12)	
O1	1.13942 (16)	−0.07866 (13)	0.10420 (11)	0.0407 (3)	
N1	0.86165 (19)	0.06039 (14)	0.13584 (13)	0.0333 (3)	
C1	0.7670 (2)	0.17310 (17)	0.13529 (15)	0.0330 (4)	
C2	0.5687 (2)	0.14945 (18)	0.13814 (16)	0.0389 (4)	
H2	0.4929	0.0588	0.1347	0.047*	
C3	0.4832 (3)	0.26138 (19)	0.14616 (17)	0.0442 (4)	
H3	0.3494	0.2446	0.1477	0.053*	
C4	0.5911 (3)	0.39724 (19)	0.15200 (17)	0.0436 (4)	
C5	0.7878 (3)	0.41810 (19)	0.14396 (18)	0.0479 (5)	
H5	0.8627	0.5082	0.1452	0.057*	
C6	0.8752 (3)	0.30679 (19)	0.13413 (18)	0.0435 (4)	
H6	1.0071	0.3221	0.1267	0.052*	
C7	0.4985 (3)	0.5211 (2)	0.1706 (2)	0.0656 (6)	
H7A	0.3635	0.4877	0.1285	0.098*	
H7C	0.5673	0.5921	0.1321	0.098*	
H7B	0.5062	0.5619	0.2629	0.098*	
C8	0.8407 (2)	0.00365 (18)	0.23459 (16)	0.0362 (4)	
H8	0.7570	0.0334	0.2897	0.043*	
C9	0.9333 (2)	−0.09941 (17)	0.26675 (15)	0.0348 (4)	
C10	0.8838 (3)	−0.16148 (18)	0.37372 (16)	0.0378 (4)	
C11	0.7251 (3)	−0.1453 (2)	0.43815 (17)	0.0472 (5)	
H11	0.6482	−0.0889	0.4136	0.057*	
C12	0.6802 (3)	−0.2103 (2)	0.53611 (18)	0.0582 (5)	
H12	0.5739	−0.1976	0.5768	0.070*	
C13	0.7941 (3)	−0.2955 (2)	0.57493 (19)	0.0631 (6)	
H13	0.7626	−0.3406	0.6406	0.076*	
C14	0.9498 (3)	−0.3120 (2)	0.51683 (19)	0.0562 (5)	
H14	1.0257	−0.3677	0.5442	0.067*	
C15	1.0000 (3)	−0.24677 (19)	0.41566 (17)	0.0436 (4)	
C16	1.1607 (3)	−0.2661 (2)	0.35237 (19)	0.0494 (5)	
H16	1.2390	−0.3194	0.3817	0.059*	
C17	1.2044 (3)	−0.2103 (2)	0.25140 (18)	0.0454 (4)	
H17	1.3110	−0.2259	0.2125	0.054*	
C18	1.0881 (2)	−0.12706 (17)	0.20317 (16)	0.0359 (4)	

### Atomic displacement parameters (Å^2^)

**Table:**

	*U*^11^	*U*^22^	*U*^33^	*U*^12^	*U*^13^	*U*^23^
Ni1	0.02674 (17)	0.03779 (19)	0.03807 (18)	0.01097 (13)	0.01075 (12)	0.01204 (13)
O1	0.0334 (6)	0.0540 (8)	0.0446 (7)	0.0189 (6)	0.0146 (5)	0.0208 (6)
N1	0.0292 (7)	0.0346 (7)	0.0391 (7)	0.0109 (6)	0.0093 (6)	0.0112 (6)
C1	0.0320 (9)	0.0351 (9)	0.0329 (8)	0.0120 (7)	0.0086 (7)	0.0064 (7)
C2	0.0355 (9)	0.0368 (9)	0.0456 (10)	0.0102 (8)	0.0143 (8)	0.0087 (8)
C3	0.0352 (10)	0.0480 (11)	0.0519 (10)	0.0181 (8)	0.0145 (8)	0.0073 (9)
C4	0.0496 (11)	0.0419 (10)	0.0409 (9)	0.0222 (9)	0.0060 (8)	0.0045 (8)
C5	0.0498 (11)	0.0345 (10)	0.0569 (11)	0.0072 (8)	0.0047 (9)	0.0132 (9)
C6	0.0316 (9)	0.0440 (10)	0.0553 (11)	0.0093 (8)	0.0070 (8)	0.0149 (9)
C7	0.0751 (15)	0.0541 (13)	0.0754 (14)	0.0374 (12)	0.0151 (12)	0.0102 (11)
C8	0.0318 (9)	0.0382 (9)	0.0382 (9)	0.0088 (7)	0.0101 (7)	0.0077 (8)
C9	0.0320 (9)	0.0359 (9)	0.0359 (9)	0.0075 (7)	0.0057 (7)	0.0102 (7)
C10	0.0403 (10)	0.0350 (9)	0.0341 (9)	0.0047 (7)	0.0044 (7)	0.0075 (7)
C11	0.0482 (11)	0.0544 (12)	0.0406 (10)	0.0123 (9)	0.0106 (8)	0.0154 (9)
C12	0.0610 (13)	0.0704 (14)	0.0441 (11)	0.0107 (11)	0.0184 (10)	0.0189 (10)
C13	0.0860 (17)	0.0624 (14)	0.0445 (11)	0.0130 (12)	0.0165 (11)	0.0255 (10)
C14	0.0778 (15)	0.0503 (12)	0.0444 (11)	0.0190 (11)	0.0083 (10)	0.0196 (10)
C15	0.0533 (11)	0.0366 (10)	0.0379 (9)	0.0089 (8)	0.0030 (8)	0.0093 (8)
C16	0.0562 (12)	0.0458 (11)	0.0521 (11)	0.0236 (9)	0.0043 (9)	0.0166 (9)
C17	0.0424 (10)	0.0500 (11)	0.0505 (11)	0.0215 (9)	0.0107 (8)	0.0156 (9)
C18	0.0317 (9)	0.0360 (9)	0.0376 (9)	0.0065 (7)	0.0038 (7)	0.0086 (8)

### Geometric parameters (Å, °)

**Table:**

Ni1—O1^i^	1.8255 (11)	C7—H7B	0.9600
Ni1—O1	1.8255 (11)	C8—H8	0.9300
Ni1—N1	1.8964 (13)	C8—C9	1.427 (2)
Ni1—N1^i^	1.8964 (13)	C9—C10	1.447 (2)
O1—C18	1.302 (2)	C9—C18	1.402 (2)
N1—C1	1.4420 (18)	C10—C11	1.404 (2)
N1—C8	1.303 (2)	C10—C15	1.423 (2)
C1—C2	1.380 (2)	C11—H11	0.9300
C1—C6	1.381 (2)	C11—C12	1.370 (3)
C2—H2	0.9300	C12—H12	0.9300
C2—C3	1.383 (2)	C12—C13	1.399 (3)
C3—H3	0.9300	C13—H13	0.9300
C3—C4	1.379 (3)	C13—C14	1.350 (3)
C4—C5	1.381 (3)	C14—H14	0.9300
C4—C7	1.518 (2)	C14—C15	1.410 (3)
C5—H5	0.9300	C15—C16	1.416 (3)
C5—C6	1.386 (2)	C16—H16	0.9300
C6—H6	0.9300	C16—C17	1.345 (3)
C7—H7A	0.9600	C17—H17	0.9300
C7—H7C	0.9600	C17—C18	1.434 (2)
			
O1^i^—Ni1—O1	180	H7C—C7—H7B	109.5
O1^i^—Ni1—N1	88.02 (5)	C8—N1—Ni1	123.82 (10)
O1—Ni1—N1	91.98 (5)	C8—N1—C1	114.66 (13)
O1^i^—Ni1—N1^i^	91.98 (5)	C8—C9—C10	120.43 (14)
O1—Ni1—N1^i^	88.02 (5)	C9—C8—H8	116.7
O1—C18—C9	124.55 (14)	C9—C18—C17	118.85 (15)
O1—C18—C17	116.56 (14)	C10—C11—H11	119.0
N1—Ni1—N1^i^	180)	C11—C10—C9	124.15 (15)
N1—C8—H8	116.7	C11—C10—C15	117.29 (16)
N1—C8—C9	126.64 (14)	C11—C12—H12	120.0
C1—N1—Ni1	121.52 (10)	C11—C12—C13	120.04 (19)
C1—C2—H2	120.2	C12—C11—C10	122.01 (17)
C1—C2—C3	119.58 (16)	C12—C11—H11	119.0
C1—C6—C5	120.18 (16)	C12—C13—H13	120.1
C1—C6—H6	119.9	C13—C12—H12	120.0
C2—C1—N1	120.54 (15)	C13—C14—H14	119.2
C2—C1—C6	119.37 (14)	C13—C14—C15	121.60 (18)
C2—C3—H3	119.1	C14—C13—C12	119.80 (19)
C3—C2—H2	120.2	C14—C13—H13	120.1
C3—C4—C5	117.94 (15)	C14—C15—C10	119.25 (17)
C3—C4—C7	121.20 (17)	C14—C15—C16	121.88 (16)
C4—C3—C2	121.79 (16)	C15—C10—C9	118.55 (15)
C4—C3—H3	119.1	C15—C14—H14	119.2
C4—C5—H5	119.5	C15—C16—H16	118.8
C4—C5—C6	121.00 (17)	C16—C15—C10	118.86 (16)
C4—C7—H7A	109.5	C16—C17—H17	119.7
C4—C7—H7C	109.5	C16—C17—C18	120.68 (16)
C4—C7—H7B	109.5	C17—C16—C15	122.48 (15)
C5—C4—C7	120.84 (18)	C17—C16—H16	118.8
C5—C6—H6	119.9	C18—O1—Ni1	127.72 (10)
C6—C1—N1	120.08 (14)	C18—C9—C8	118.71 (15)
C6—C5—H5	119.5	C18—C9—C10	120.46 (14)
H7A—C7—H7C	109.5	C18—C17—H17	119.7
H7A—C7—H7B	109.5		

Symmetry codes: (i) −*x*+2, −*y*, −*z*.
